# Robust Evaluation of Ultraviolet-C Sensitivity for SARS-CoV-2 and Surrogate Coronaviruses

**DOI:** 10.1128/Spectrum.00537-21

**Published:** 2021-10-20

**Authors:** S. J. Boegel, M. Gabriel, M. Sasges, B. Petri, M. R. D’Agostino, A. Zhang, J. C. Ang, M. S. Miller, S. M. Meunier, M. G. Aucoin

**Affiliations:** a Department of Chemical Engineering, University of Waterloo, Waterloo, Canada; b Trojan Technologies, London, Canada; c Michael G. DeGroote Institute for Infectious Disease Research, McMaster Immunology Research Centre, Department of Biochemistry and Biomedical Sciences, McMaster University, Hamilton, Ontario, Canada; University of Arizona

**Keywords:** coronavirus, ultraviolet, disinfection, SARS-CoV-2, HCoV-229E, HCoV-OC43, MHV, T1 bacteriophage, collimated beam, irradiation, HuCoV 229E, HuCoV OC43, baculovirus vector

## Abstract

UV light, more specifically UV-C light at a wavelength of 254 nm, is often used to disinfect surfaces, air, and liquids. In early 2020, at the cusp of the COVID-19 pandemic, UV light was identified as an efficient means of eliminating coronaviruses; however, the variability in published sensitivity data is evidence of the need for experimental rigor to accurately quantify the effectiveness of this technique. In the current study, reliable and reproducible UV techniques have been adopted, including accurate measurement of light intensity, consideration of fluid UV absorbance, and confirmation of uniform dose delivery, including dose verification using an established biological target (T1UV bacteriophage) and a resistant recombinant virus (baculovirus). The experimental results establish the UV sensitivity of SARS-CoV-2, HCoV-229E, HCoV-OC43, and mouse hepatitis virus (MHV) and highlight the potential for surrogate viruses for disinfection studies. All four coronaviruses were found to be easily inactivated by 254 nm irradiation, with UV sensitivities of 1.7, 1.8, 1.7, and 1.2 mJ/cm^2^/log_10_ reduction for SARS-CoV-2, HCoV-229E, HCoV-OC43, and MHV, respectively. Similar UV sensitivities for these species demonstrate the capacity for HCoV-OC43, HCoV-229E, and MHV to be considered surrogates for SARS-CoV-2 in UV-inactivation studies, greatly reducing hazards and simplifying procedures for future experimental studies.

**IMPORTANCE** Disinfection of SARS-CoV-2 is of particular importance due to the global COVID-19 pandemic. UV-C irradiation is a compelling disinfection technique because it can be applied to surfaces, air, and water and is commonly used in drinking water and wastewater treatment facilities. UV inactivation depends on the dose received by an organism, regardless of the intensity of the light source or the optical properties of the medium in which it is suspended. The 254 nm irradiation sensitivity was accurately determined using benchmark methodology and a collimated beam apparatus for four coronaviruses (SARS-CoV-2, HCoV-229E, HCoV-OC43, and MHV), a surrogate indicator organism (T1UV), and a resistant recombinant virus (baculovirus vector). Considering the light distribution across the sample surface, the attenuation of light intensity with fluid depth, the optical absorbance of the fluid, and the sample uniformity due to mixing enable accurate measurement of the fundamental inactivation kinetics and UV sensitivity.

## INTRODUCTION

SARS-CoV-2 has been detected by real-time reverse transcription-PCR (RT-PCR) in fecal samples (1) and in municipal wastewater (2), and transmission associated with sewage aerosols was documented during the 2003 outbreak of the closely related SARS-CoV coronavirus (3). Recently, SARS-CoV-2 was detected in groundwater, rivers, and dam water in the Monterrey, Mexico, region (4). These observations raise critical questions about the adequacy of treatment for drinking water and wastewater. UV is one of the primary disinfection techniques used for drinking water and wastewater in North America, yet its efficacy against coronaviruses, and SARS-CoV-2 in particular, remains unclear. For example, a literature review by Hessling et al. (5) found published UV sensitivity values for coronaviruses ranging from 1.5 to 40.5 mJ/cm^2^/log_10_ reduction and even as high as 11,000 mJ/cm^2^/log_10_ reduction when evaluated in optically absorbing media.

Many studies of UV inactivation fail to adequately consider the optics of the experimental arrangement and of the fluid suspension, causing UV dose to be evaluated incorrectly. As Hessling points out, researchers often use fluid suspensions with high optical absorbance, such as cell culture media, but do not consider the strong attenuation by these media. Some investigators use extended arc lamps as UV sources and locate the fluid samples close to the lamp. As a result, UV-C arriving at the samples off-axis is not measured correctly because the light is reflected, refracted, or absorbed by the sample containers and the liquid/air interface. These and other factors have led to considerable variability in reported inactivation kinetics. Therefore, there is an urgent need for a rigorous evaluation of the UV sensitivity of SARS-CoV-2 and other coronaviruses.

In this study, the UV-C disinfection kinetics for SARS-CoV-2 and three other coronaviruses in aqueous suspension are measured experimentally. Furthermore, a virus of known UV sensitivity, T1UV bacteriophage, and a known UV-resistant recombinant virus, baculovirus, were evaluated using the same methods and equipment, ensuring that these data can be reliably compared with those from other studies.

## RESULTS

### SARS-CoV-2.

**FIG 1:**
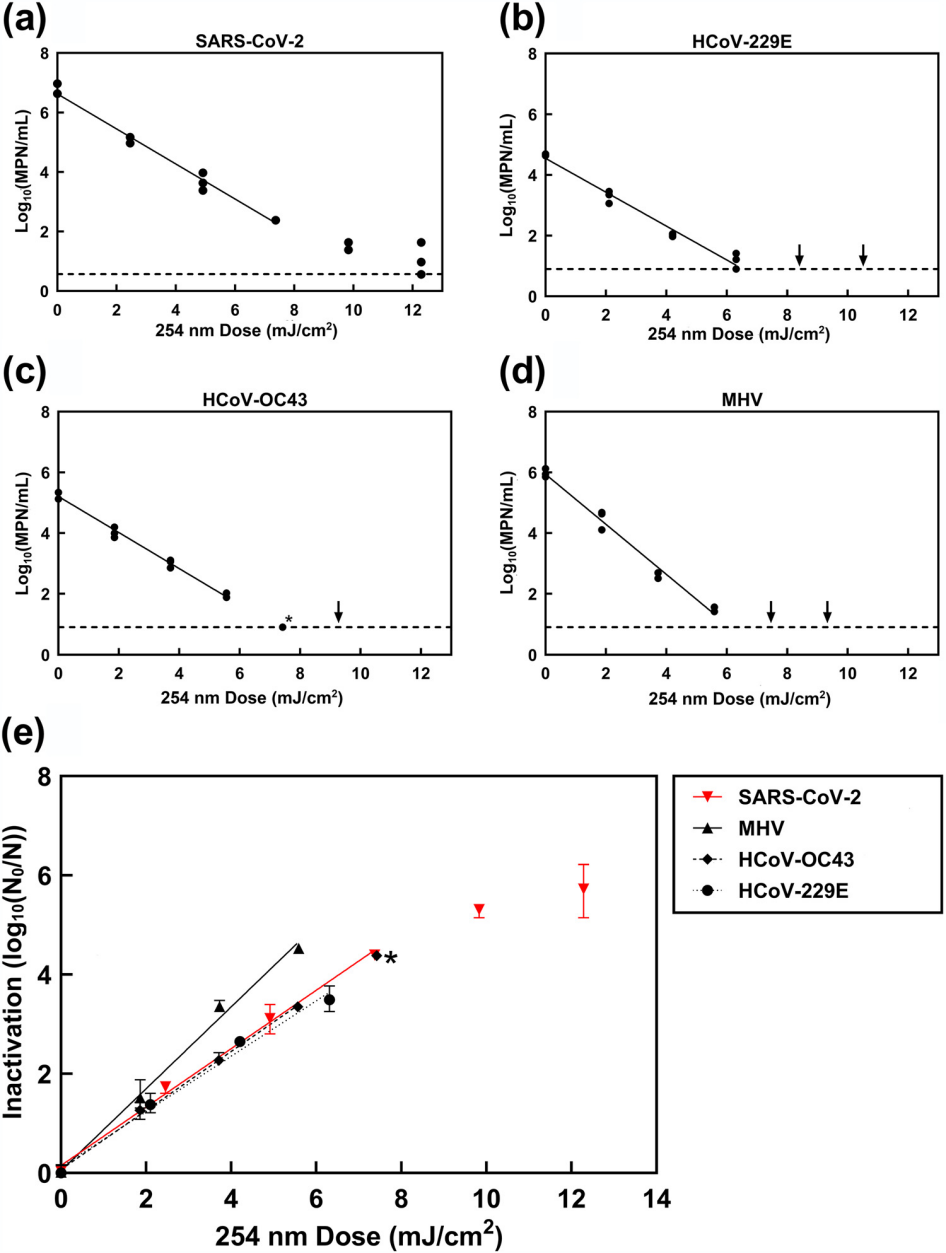
Inactivation of SARS-CoV-2 and three surrogate coronaviruses by exposure to UV-C irradiation. Doses are corrected for an adjustment factor for each radiometer. *, 1 replicate no virus detected and 2 replicates at detection limit (HCoV-OC43); point excluded from fitted line. SARS-CoV-2: *y* = 0.59*x* + 0.15, *R*^2^ = 0.99; HCoV-229E: *y* = 0.56*x* + 0.12, *R*^2^ = 0.99; HCoV-OC43: *y* = 0.59*x* + 0.07, *R*^2^ = 0.99; MHV: *y* = 0.82*x* + 0.05, *R*^2^ = 0.99. (a to d) Triplicate irradiations were performed for each dose; all replicates shown on plot. Dashed lines represent detection limits. Arrows represent no virus detected in assay at those doses. (e) Each point represents the mean of triplicate sample irradiation. Error bars represent range of data. Detection limits were 6.22, 5.09, 4.38, and 3.77 log_10_(N_0_/N) for SARS-CoV-2, MHV, HCoV-OC43, and HCoV-229E, respectively.

SARS-CoV-2 was found to be sensitive to UV-C irradiation. Starting from a SARS-CoV-2 suspension with a titer of approximately 6 × 10^6^ most probable number (MPN)/ml, the 254 nm inactivation was first order over 4.5 logs of inactivation, with a slope of 0.59 log_10_ reduction cm^2^/mJ. This is equivalent to a UV sensitivity of 1.7 mJ/cm^2^/log_10_ reduction. There was some tailing in the data at high doses where measured virus titer approached the limit of detection, with decreasing slope at extreme dose (Fig. 1a).

### Surrogate coronaviruses.

Coronaviruses HCoV-229E, HCoV-OC43, and MHV were also irradiated with UV-C and inoculated onto confluent cell monolayers, and their surviving infectious titers were quantified. All three viruses exhibited first-order kinetics over at least 3.5 logs of inactivation. Higher UV-C doses resulted in no virus detected in the assays. Fitted lines had slopes of 0.56, 0.59, and 0.82 log_10_ reduction cm^2^/mJ, which is equivalent to doses required per log_10_ inactivation of 1.8, 1.7, and 1.2 mJ/cm^2^ for HCoV-229E, HCoV-OC43, and MHV, respectively (Fig. 1).

### T1 bacteriophage.

**FIG 2:**
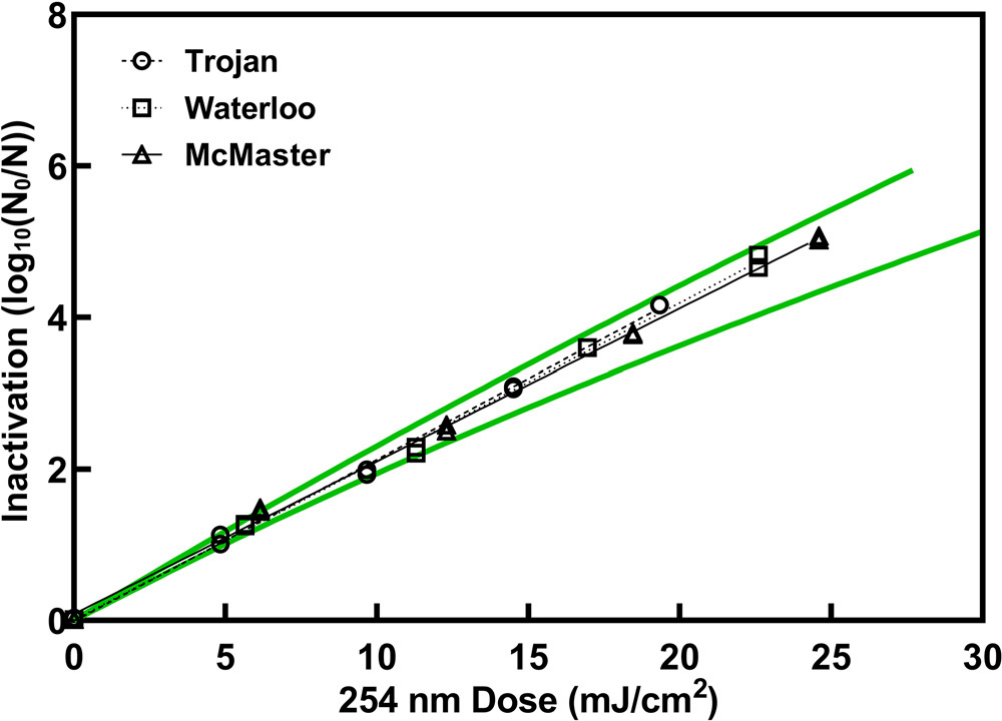
Verification of collimated beam dose delivery from T1UV bacteriophage. Dose response data generated with collimated beam kits used at both the University of Waterloo and McMaster University, as well as a third collimated beam kit at a reference laboratory (Trojan Technologies). For each collimated beam kit, duplicate irradiations were performed for each dose, and values shown are averages of duplicate plating of each irradiated sample. Doses are corrected for an adjustment factor for each radiometer. All dose-response data fell within the 95th percentile prediction intervals for T1UV, shown as solid lines (6).

The UV sensitivity of T1UV bacteriophage (HER 468, GAP EnviroMicrobial Services, London, ON, Canada) was determined to be 4.8 mJ/cm^2^/log_10_ reduction (Fig. 2), which is within the 95th percentile prediction intervals calculated for 166 dose-response data sets for T1UV (6), indicating that irradiance measurements, optical absorbance measurements, and geometric factors were all properly accounted for in determining dose accurately for all three units.

### Baculovirus vector.

**FIG 3:**
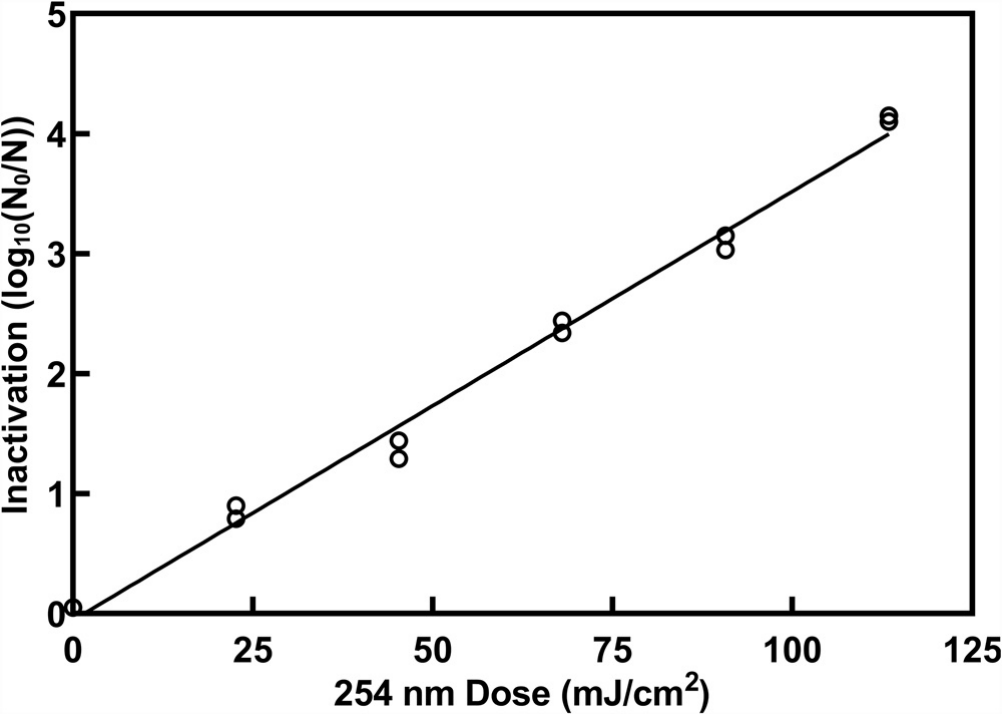
Inactivation of baculovirus by exposure to doses of UV-C irradiation. Doses are corrected for an adjustment factor for each radiometer. Duplicate irradiations were performed for each dose and all replicates are shown on the plot; *y* = 0.036*x* − 0.06, *R*^2^ = 0.99.

The UV sensitivity of a more resistant enveloped recombinant virus that carries a double-stranded DNA genome was determined to provide an additional validation of the relatively high measured sensitivities of the coronaviruses. The recombinant virus utilized was the budded form of baculovirus (Autographa californica multiple nucleopolyhedrovirus) and was irradiated using the same collimated beam apparatus and irradiance sensors as those used for the coronaviruses. The baculovirus exhibited first-order kinetics over 4 logs of inactivation, with the fitted line having a slope of 0.036 log_10_ reduction cm^2^/mJ. This corresponds to a UV sensitivity of 27.8 mJ/cm^2^/log_10_ reduction (Fig. 3).

## DISCUSSION

### SARS-CoV-2.

SARS-CoV-2 is highly susceptible to 254 nm UV irradiation, like other coronaviruses that can be used as suitable surrogates for this highly pathogenic virus. For context, common disinfection targets, human adenovirus and poliovirus, are both significantly more resistant to UV inactivation, with average 254 nm UV sensitivities of 42.3 and 8.0 mJ/cm^2^/log_10_ reduction, respectively (7).

Despite the common use of UV as a means for water disinfection, available reports show a remarkable range in the susceptibility of SARS-CoV-2 to UV irradiation. UV encompasses light in a range of wavelengths and is further classified as UV-A (315 to 400 nm), UV-B (280 to 315 nm), or UV-C (100 to 280 nm). The primary mode of UV inactivation of organisms is through the disruption of their genetic material, particularly through the formation of pyrimidine dimers (8), initiated by absorption of UV photons. Peak absorption by nucleic acids is around 260 nm. Typically, irradiations are performed at 254 nm using a mercury vapor lamp; however, with the emergence of light emitting diodes (LEDs) that emit at different wavelengths, care should be taken when reporting the susceptibility of organisms, and viruses, as the inactivation is a function of the specific wavelength.

The inactivation is also a function of the ability to reach the target; therefore, the placement of the sample with respect to the light source and the medium in which the organism/virus resides also play a role in understanding the susceptibility of the organism/virus to UV. Optical absorbance of the medium will reduce the intensity of the UV light in the fluid, thereby decreasing the inactivation. Serum, a component commonly used in cell culture for the propagation of viruses, has a very high optical absorbance and is one cause of lower-than-actual UV-sensitivity reporting. Additionally, phenol red is typically added to media as a pH indicator and also has an effect on optical transmittance, although this effect is less significant than that of serum (Table 1).

**TABLE 1:** Measured UV transmittance (UVT) of cell culture media formulations containing various concentrations of phenol red and FBS; UVT of media was measured using a Real UV254 P200 photometer at ambient temperature and a pH of 7.4

Formulation	Base medium	Phenol red (mg/liter)^*a*^	FBS (%, vol/vol)	Measured UVT (%)
1	RPMI 1640	0	0	34.6
2	RPMI 1640	0	2	19.4
3	RPMI 1640	0	10	1.5
4	RPMI 1640	5	0	30.2
5	RPMI 1640	5	2	18.9
6	RPMI 1640	5	10	1.4
7	EMEM	0	0	34.2
8	EMEM	0	2	20.4
9	EMEM	0	10	1.7
10	EMEM	10	0	25.5
11	EMEM	10	2	14.0
12	EMEM	10	10	0.7

a Standard phenol red concentration is 5 mg/liter in RPMI 1640 and 10 mg/liter in EMEM.

For example, although Storm et al. (9) found that SARS-CoV-2 could be readily inactivated with UV-C (they found first-order inactivation of infectivity with a UV sensitivity of about 1.8 mJ/cm^2^/log_10_ reduction over a 2-log inactivation), their approach and methodology were nonstandard, making it hard to decipher if they had properly found the intrinsic kinetics of inactivation. Not only did the medium contain fetal bovine serum (FBS; 2%) and phenol red, but the irradiated liquid was kept as a liquid droplet on a clean surface. Though the volume was minute (5 μl), given the high absorbance and the lack of fluid mixing, it is hard to state that all elements within the fluid were exposed to the same amount of irradiation.

Similarly, Biasin et al. (10) used an unconventional setup, with samples placed only 3 cm from the mercury lamp and not stirred. The former can result in light not penetrating the liquid but reflecting away from the sample, and like the previous study, the lack of mixing does not ensure that uniform fluence is applied. More specifically, strongly off-axis radiation may be reflected from the liquid/air interface and may be affected (absorbed or reflected) by the sample container. Regardless, the UV sensitivity values for different initial concentrations ranged from approximately 0.5 to 7.5 mJ/cm^2^/log_10_ reduction, spanning our estimate of UV sensitivity. The range of log_10_ reduction may also be the result of the infectivity assay methodology of measuring the amount of virus amplified after irradiation as opposed to the number of viruses that remain after irradiation.

These issues are further exemplified in the report from Heilingloh et al. (11), which found a much higher resistance, with a calculated UV sensitivity for SARS-CoV-2 of about 98 mJ/cm^2^/log_10_ reduction. This may again be explained by the absorbance of the medium and lack of fluid mixing. In their work, virus was kept in Dulbecco’s modified Eagle’s medium (DMEM) cell culture medium with 10% FBS. Though they did not report absorbance, we have measured this formulation to have an extremely high absorbance of about 240 m^−1^. For a sample depth of 3 mm (considering 600 μl aliquots in 24-well plate format), the intensity at the bottom of an unstirred sample would be about 11% of that at the surface. As a result, virions at the bottom of the sample would dominate the surviving numbers after irradiation due to the reduced UV intensity and lead to artificially low sensitivity values.

Patterson et al. (12) used a multi-254 nm lamp UV curing device to generate UV dose-response curves for SARS-COV-2. We estimated their UV sensitivities based on tissue culture infectious dose (TCID) and plaque assays to be 6.7 and 5.3 mJ/cm^2^/log_10_ reduction, respectively. However, their device did not have collimated light and it was not stated where the in-built intensity sensor was in relation to the samples. In addition, samples were not stirred and the absorbance of the medium (DMEM with 4% FBS) was not accounted for, leading to a calculated UV resistance that is higher than the actual viral UV resistance.

Inagaki et al. (13) irradiated CoV-2 using LEDs with peak emission at 282 nm. The absorbance of RNA and DNA at 282 nm is about 56% of that at 254 nm (14), so we would expect that inactivation at 282 nm would be about half that at 254 nm. Inagaki reported a 3 log_10_ (99.9%) inactivation from a dose of 37.5 mJ/cm^2^, corresponding to a UV sensitivity of 12.5 mJ/cm^2^/log_10_ reduction at 282 nm. This would roughly correspond to 6 mJ/cm^2^/log_10_ reduction at 254 nm. In their study, the irradiation distance was only 2 cm, samples were not stirred, and the optical absorbance of the virus suspension was not determined. These factors all tend to result in a higher reported UV resistance.

Our results are broadly consistent with the literature on SARS-CoV-2 even though many of the previously reported numbers do not reflect the true sensitivity of SARS-CoV-2. Given the global pandemic, and a need to find ways to eliminate SARS-CoV-2, it is not surprising that consistent and robust methodologies were not prioritized. Our focus on robust methodology and controls results in accurate UV-sensitivity and inactivation kinetics data.

### Comparison with SARS.

Between 2002 and 2004, an outbreak of severe acute respiratory syndrome (SARS) caused by the SARS-coronavirus (CoV) dominated the headlines, and by 2004, reports on UV inactivation of this virus were published (5). SARS-CoV is closely related to the SARS-CoV-2 virus. Two studies produced data that could be used to determine UV sensitivity at 254 nm. Kariwa et al. (15) reported inactivation from a starting titer of 7.6 log_10_(TCID_50_/ml) to 2.3 log_10_(TCID_50_/ml) after a reported dose of 120 mJ/cm^2^, which corresponds to a UV sensitivity of 22 mJ/cm^2^/log_10_ reduction. Darnell et al. (16) measured a higher resistance, reducing a starting titer of about 5.8 log_10_(TCID_50_/ml) to 1 log_10 (_TCID_50_/ml) after a calculated dose of 1,445 mJ/cm^2^, which corresponds to a UV sensitivity of 300 mJ/cm^2^/log_10_ reduction. Both studies report values that are not in line with those of SARS-CoV-2 or other coronaviruses and fail to describe the geometry of the irradiation system, sample stirring, or absorbance measurements. These aspects are critical given that viruses were in minimum essential medium (MEM) cell culture medium with 10% FBS in the Kariwa et al. study and in DMEM cell culture medium with 10% FBS in the Darnell et al. study. Furthermore, without mixing, minimal light penetration would occur for sample depths of 3 mm (15) and 1 cm (16). All these factors tend to cause an apparent UV resistance to be determined that is higher than the actual UV resistance.

UV-inactivation studies are prominent in the advent of an adverse event (such as an outbreak or a pandemic) because there is a body of knowledge that supports the use of UV for inactivating these pathogens. However, UV encompasses a wide range of light wavelengths, and the effect on targets has as much to do with the light source as it does the overall setup that enables the light to reach the target. Much of what has initially been reported for SARS-CoV-2 suffers from the same issues that can be identified in the studies on SARS-CoV. Our group has previously commented on these issues for a different application, the susceptibility of adventitious agents in the biopharmaceutical industry (7). In the current work, we report the sensitivity of SARS-CoV-2 and highlight the best practices to ensure that future research reports appropriate sensitivities for the pathogen of concern.

### Comparison with other coronaviruses.

Coronaviruses are single-stranded RNA viruses with genomes that vary from 26 kB to 32 kB and share significant similarities (17). Other than the above-mentioned pandemic viruses, several less-pathogenic coronaviruses have become endemic in our society. These viruses, which lead to less-severe outcomes, are also prime candidates as surrogates for SARS-CoV-2 in research laboratories. In this work, we show that HCoV-229E, HCoV-OC43, and MHV all have susceptibility to 254 nm UV similar to that of SARS-CoV-2.

Together, Gerchman et al. (18) and Ma et al. (19) have investigated the UV sensitivity of these 3 coronaviruses. Using LEDs, Gerchman et al. (18) evaluated the inactivation of HCoV-OC43 at several wavelengths, not including 254 nm. The closest wavelength to 254 nm that was used in that study was 267 nm. Although the samples were not stirred, the optical absorbance of the virus suspension was very low (98% UV transmittance [UVT] at 1 cm), which reduces light attenuation within the sample. From the slope of their log_10_ inactivation versus dose at that wavelength, the sensitivity of HCoV-OC43 was calculated to be 2 mJ/cm^2^/log_10_ reduction, which is in good agreement with the results of the current study.

Ma et al. (19) evaluated the UV-C sensitivity of the other two coronaviruses evaluated in the current study, HCoV-229E and MHV. Their approach used a collimated beam apparatus, stirred samples, and accounted for the optical absorbance of the virus suspension. They reported rate constants at 254 nm for HCoV-229E and MHV of 0.59 and 0.93 cm^2^/mJ, corresponding to 1.7 and 1.1 mJ/cm^2^/log_10_ reduction, respectively, which are also consistent with the results reported herein.

### Coronavirus sensitivity and the absorption spectrum of nucleic acids.

**FIG 4:**
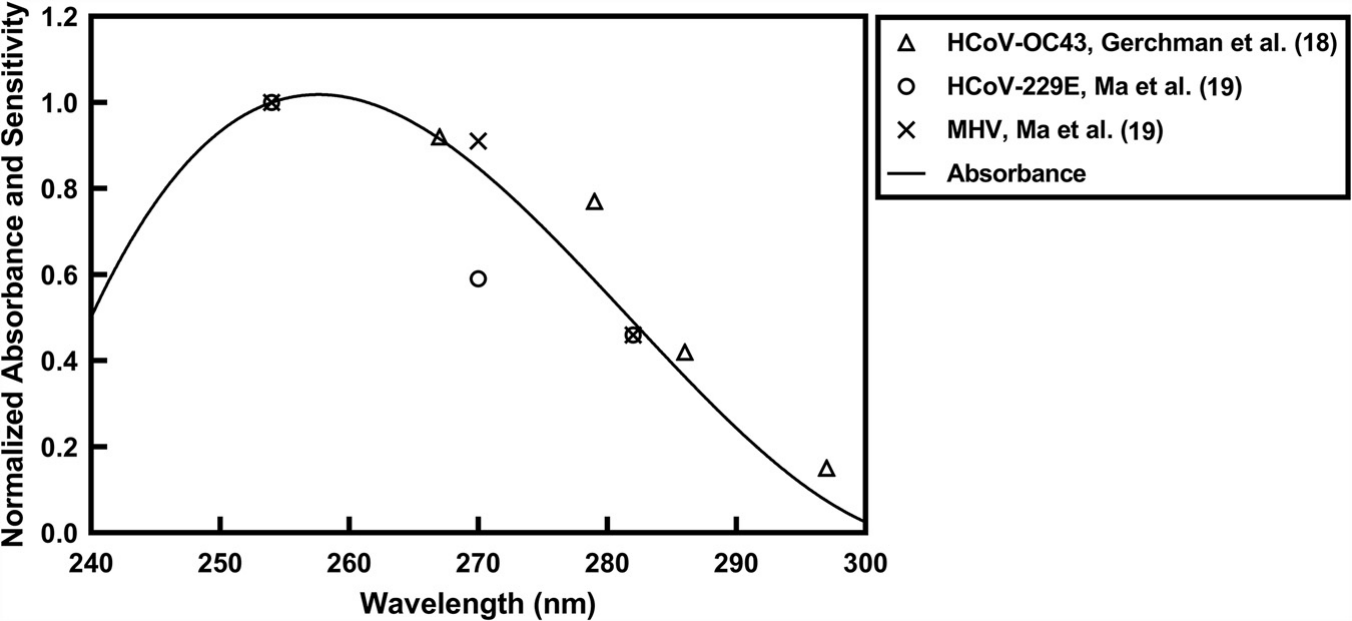
Absorption spectrum of RNA and DNA, along with measured sensitivity values for three coronaviruses: HCoV-OC43, HCoV-229E, and MHV. All values normalized to 254 nm.

Given that both Gerchman et al. and Ma et al. evaluated the inactivation of coronaviruses at several UV wavelengths, and their results were comparable to ours, it is fair to examine the impact of the wavelength on UV sensitivity from their data. To obtain the UV sensitivities at different wavelengths from Gerchman et al., we extracted the log_10_ inactivation data (over the first 3 logs of inactivation) versus dose and calculated the slope of the line through the origin, yielding the UV sensitivity at that wavelength. Ma et al. reported the sensitivity values directly. We then normalized the sensitivity values to unity at 254 nm by comparing with the absorption spectrum of DNA and RNA (14, 20) (Table 2 and Fig. 4). Since Gerchman et al. did not have values at 254 nm, the sensitivity value at 267 nm was set at 0.92, which is the relative absorbance of nucleic acids at 267 nm compared to 254 nm. The UV sensitivity values as a function of wavelength correspond closely to the absorption spectrum of DNA and RNA (Fig. 4), which is consistent with the known primary mechanism of UV disinfection: the formation of cyclobutane pyrimidine dimers, requiring the absorption of a photon. This correlation may allow the prediction of UV effectiveness of other monochromatic sources, such as LEDs, at wavelengths not already measured.

**TABLE 2:** UV inactivation results for surrogate coronaviruses calculated based on data from Gerchman et al. (18) and as reported by Ma et al. (19) with sensitivity normalized to unity at 254 nm

Wavelength (nm)	HCoV-OC43, Gerchman et al. (18)		HCoV-229E, Ma et al. (19)		MHV, Ma et al. (19)	
	Dose/log10 (mJ/cm^2^)	Normalized sensitivity	Dose/log10 (mJ/cm^2^)	Normalized sensitivity	Dose/log10 (mJ/cm^2^)	Normalized sensitivity
254			1.7	1	1.1	1
267	2.0	0.92				
270			2.9	0.59	1.2	0.91
279	2.4	0.77				
282			3.7	0.46	2.3	0.46
286	4.3	0.42				
297	12.0	0.15				

### HCoV-229E and HCoV-OC43 are candidate surrogates for SARS-CoV-2 in UV-C irradiation.

The results presented in this study show that SARS-CoV-2 is particularly sensitive to 254 nm light, with a UV sensitivity of 1.7 mJ/cm^2^/log_10_ reduction compared to the other coronaviruses HCoV-229E, HCoV-OC43, and MHV that have UV sensitivities of 1.8, 1.7, and 1.2 mJ/cm^2^/log_10_ reduction, respectively. The experimental methodologies followed in this study have been proven to be robust and reproducible based on the UV sensitivity determined for the widely characterized bacteriophage T1UV and a well-characterized UV-resistant virus, baculovirus vector. In these studies, a collimated beam apparatus was used and both light intensity and fluid optical transmittance were considered to ensure that the dose delivered to the organism was evaluated precisely. The close agreement between the inactivation kinetics for SARS-CoV-2, HCoV-229E, and HCoV-OC43 indicate that the latter two viruses are good candidates for surrogates for SARS-CoV-2 in UV-C irradiation studies.

## MATERIALS AND METHODS

### Viruses and cell cultures.

SARS-CoV-2 isolate SB3-TYAGNC (21) was provided by Arinjay Banerjee, Karen Mossman, and Samira Mubareka and was propagated on VERO C1008 (Vero 76, clone E6, Vero E6; ATCC CRL1586) cells under containment level 3 (CL 3) containment (as per the Canadian Human Pathogen and Toxin Act). Vero E6 cells were maintained in Dulbecco’s modified Eagle’s medium (DMEM; Thermo Fisher Scientific, Waltham, MA, USA) supplemented with 10% (vol/vol) fetal bovine serum (FBS), penicillin-streptomycin, and l-glutamine (Thermo Fisher Scientific). SARS-CoV-2 was cultured, irradiated, and quantified in the CL3 laboratory at McMaster University (Hamilton, Ontario).

Three other coronaviruses were cultured, irradiated, and quantified at the University of Waterloo. Human coronavirus 229E (HCoV-229E; ATCC VR-740), human coronavirus OC43 (HCoV-OC43; betacoronavirus 1 ATCC VR-1588), and murine hepatitis virus (MHV; ATCC VR-764) were propagated on MRC-5 (ATCC CCL-171), HCT-8 (HRT-18; ATCC CCL-244), and NCTC clone 1469 (derivative of NCTC 721; ATCC CCL-9.1) cells, respectively (Cedarlane, Burlington, ON, Canada) under CL2 containment (as per the Canadian Human Pathogen and Toxin Act). MRC-5 cells were maintained in Eagle’s minimum essential medium (EMEM) (Wisent BioProducts, Saint-Jean-Baptiste, QC, Canada) supplemented with 10% (vol/vol) FBS (Wisent BioProducts). HCT-8 cells were maintained in RPMI 1640 medium (Wisent BioProducts) supplemented with 10% (vol/vol) FBS. NCTC 1469 cells were maintained in Dulbecco’s modified Eagle’s medium (DMEM) (Wisent BioProducts) supplemented with 10% (vol/vol) horse serum (Sigma-Aldrich, Oakville, ON, Canada). Cells were serially passaged in cell culture-treated T-flasks (Thermo Fisher Scientific) and incubated at 37°C in a 5% CO_2_ in air atmosphere.

SARS-CoV-2 was propagated on Vero E6 cells by infection at a multiplicity of infection (MOI) of 0.01 with a minimal volume of serum-free DMEM. After a 1-h incubation at 34°C, infectious medium was removed and replaced with 30 ml DMEM plus 2% (vol/vol) FBS. Virus was harvested after a 3-day incubation in a 5% CO_2_ in air atmosphere at 34°C. Supernatant was centrifuged at 1,000 × *g* and stored at −80°C.

For propagation of other coronaviruses, the appropriate cells were seeded to achieve an 80 to 90% confluent monolayer after 24 to 48 h, washed with Dulbecco’s phosphate buffered saline (D-PBS), and infected at a multiplicity of infection (MOI) of 0.01 with a minimal volume of infection medium. After 1 to 2 h of incubation, infection medium was added to reach normal working volume (e.g., 25 ml in a 175-cm^2^ tissue culture flask). The flask was then incubated until cytopathic effects were observed to have progressed through approximately 80% of the cell monolayer and the supernatant was harvested. Supernatant was centrifuged at 1,000 × *g* to remove cells and cell debris and then aliquoted and stored at −80°C until use (working stock). Flasks were incubated in a 5% CO_2_ in air atmosphere at 34°C, 33°C, and 37°C for HCoV-229E, HCoV-OC43, and MHV, respectively. Duration of incubation was 5 days, 8 days, and 22 h for HCoV-229E, HCoV-OC43, and MHV, respectively. Infection media were EMEM plus 2% (vol/vol) FBS, RPMI 1640 plus 2% (vol/vol) FBS, and NCTC-135 (Sigma-Aldrich) plus 10% (vol/vol) horse serum for HCoV-229E, HCoV-OC43, and MHV, respectively.

The baculovirus (Autographa californica multiple nucleopolyhedrovirus) vector (herein simply referred to as baculovirus) was engineered to express the fluorescent protein mKOk under the late and strong viral p10 promoter using the Bac-to-Bac baculovirus expression system (Thermo Fisher Scientific) as reported in Walji and Aucoin (22). Baculovirus was propagated on Sf-9 insect cells (Sf-9, ATCC CRL-1711). Sf-9 cells were grown in serum-free Gibco Sf-900III (Thermo Fisher Scientific). Cells were routinely maintained in 125-ml glass shake flasks (Corning GlassWorks, Corning, NY, USA) with a working volume of 30 ml at 27°C and agitated at 130 rpm. Cells were subcultured twice per week to maintain the cell density between 0.5 × 10^6^ and 5 × 10^6^ cells/ml. Cell densities were assessed using a Countess II FL automated cell counter (Invitrogen, Thermo Fisher Scientific). Cell viability was determined via the trypan blue exclusion method.

For virus propagation, a passage 2 working stock of the virus was made by infecting 1.5 × 10^6^ cells/ml of exponentially growing Sf-9 cells with recombinant virus at an MOI of 0.01. Infected cells were incubated at 27°C and agitated at 130 rpm. Cell viability was measured daily until it reached 80% (72 to 96 h postinfection), after which virus supernatant was harvested, centrifuged at 800 × *g* for 10 min, and filtered through a 0.2-μm polyethersulfone (PES) membrane (VWR International, Mississauga, ON, Canada). Purified virus was stored at 4°C until use.

Frozen virus aliquots were rapidly thawed, diluted with D-PBS, and irradiated (see below). Dilution ratios were as follows: 4 ml HCoV-229E working stock to 1 ml D-PBS, no dilution of HCoV-OC43 working stock, 1 ml MHV working stock to 4 ml D-PBS, 1 ml SARS-CoV-2 working stock to 4 ml D-PBS. Dilution ratios were chosen to achieve a high enough optical transmittance to maintain low sample treatment time while ensuring that the starting virus titer was high enough to demonstrate multilog inactivation. Each virus working stock consisted of one large multiflask batch which was harvested, clarified, and mixed to ensure homogeneity prior to aliquoting and storing at −80°C. Baculovirus samples were removed from 4°C storage and diluted 1 in 100 in D-PBS prior to irradiation.

After irradiation, infectious virus titer of samples was determined by endpoint dilution assay. Ten-fold serial dilutions of samples in the appropriate infection medium were used to inoculate 96-well plates containing confluent monolayers of the appropriate cell line. The 96-well plates were incubated in a 5% CO_2_ in air atmosphere at 37°C, 34°C, 33°C, and 37°C for SARS-CoV-2, HCoV-229E, HCoV-OC43, and MHV, respectively, and monitored for cytopathic effects. Wells were scored as either positive or negative for virus (based on presence or absence of cytopathic effects) after 5 days, 6 days, 12 days, and 4 days for SARS-CoV-2, HCoV-229E, HCoV-OC43, and MHV, respectively. Baculovirus samples were quantified using the same procedure, inoculating adherent Sf-9 cells, and incubating for 7 days at 27°C in a humidified atmosphere. Wells were scored as either positive or negative based on the presence or absence of red fluorescence when viewed under a fluorescence microscope.

Most probable number (MPN) estimation was used to quantify viral titer (23). The limit of detection was 3.61 MPN/ml for SARS-CoV-2 and 7.88 MPN/ml for all other coronaviruses. This represents the lowest calculable virus titer (i.e., one positive well in undiluted inoculum replicate wells) and differs due to the use of a larger inoculum volume per well for SARS-CoV-2.

### Collimated beam apparatuses and irradiance.

Two identical custom-made collimated beam apparatuses (Trojan Technologies, London, ON, Canada) were used to irradiate samples of virus suspension. The design followed the recommendations of Bolton and Linden (24) and utilized a low-pressure mercury vapor arc lamp emitting primarily at 253.7 nm. The lamp was contained within a housing that allowed optical emission only from an arc of approximately 5 cm length, and the lamp was positioned about 33 cm above the sample surface. This geometry ensured equivalence between measured irradiance and fluence rate (the angle-independent flux relevant for disinfection). Before use, the mercury lamp was turned on and allowed to stabilize for a minimum of 1 h.

Irradiance at the position of the fluid surface was measured using calibrated radiometers: ILT2400 and ILT1700 with SED240 detectors each equipped with a quartz W diffuser and NS254 spectral filter to ensure that only 254 nm radiation was measured (International Light Technologies, Peabody, MA, USA). Corrections were made for beam divergence, nonuniformity at the fluid surface, and reflection from the air/liquid interface.

There can be a range of readings for calibrated radiometers measuring the same light source. A best practice is to use multiple radiometers and report the average intensity (25). To accommodate experimental work in multiple labs and the necessity to sacrifice the radiometer used at the McMaster CL3 lab, a single radiometer was identified for use in each lab. To account for potential variability in measurement, an adjustment factor was calculated for each radiometer relative to the average of a pool of five calibrated radiometers determined at the start of the project on the same light source. The adjustment factors were recalculated at the end of the project to verify consistency.

### Optical transmittance.

Optical transmittance of the viral suspension at 254 nm was measured with a quartz cuvette using a Real UV254 P200 photometer (RealTech Inc, Whitby, ON, Canada) and used along with sample depth to calculate the average fluence rate in the stirred sample using the Beer-Lambert equation according to the derivation of Morowitz (26). Percent transmittance at 1 cm was measured as 12.2%, 9.3%, 9.0%, and 12.5% for the tested viral suspensions of HCoV-229E, HCoV-OC43, MHV, and SARS-CoV-2, respectively. Percent transmittance of the baculovirus suspension was 55%. Percent transmittance of the T1UV suspension was 85% for the experiment performed at Trojan Technologies and 96.4% and 96.9% for the two experiments performed at the University of Waterloo.

### UV irradiation of samples.

Five-ml samples in 10-ml beakers were irradiated while continuously stirred with a Teflon-coated stir bar actuated by a laboratory stir plate. Irradiation times were calculated for the desired dosages, following the corrections and methods for nonuniformity, divergence, reflection, and absorbance as described by Bolton and Linden (24). Irradiation times were always greater than 30 s to ensure that the fluid mixing was sufficient to achieve uniform UV exposure. Control samples were stirred in beakers for the same length of time as that which was required for the largest dose for each virus but not exposed to UV-C radiation. Triplicate irradiations were performed for each dosage and the control stirring was also performed in triplicate. Sample irradiation and control stirring were performed in a randomized order, and endpoint dilution assays were set up immediately after irradiation and collection of samples.

### UV fluence validation.

To verify the accuracy of the UV fluence delivered during irradiation, a viral clearance test was conducted with the collimated beam kits used in the two labs as well as with a third collimated beam in a reference lab (Trojan Technologies, London, ON, Canada) using T1UV bacteriophage (HER 468, GAP EnviroMicrobial Services, London, ON, Canada). T1UV is a challenge virus that has a well-characterized UV sensitivity of 5 mJ/cm^2^/log_10_ reduction due to its widespread use in numerous UV reactor bioassay challenges (6, 27). For each collimated beam kit, T1UV stock was purchased from GAP EnviroMicrobial Services and diluted 1,000-fold in PBS to a working concentration of approximately 7 log_10_(PFU/ml). Samples were irradiated at several discrete UV doses and transported on ice in coolers for next-day enumeration at GAP EnviroMicrobial Services. Enumeration of T1UV bacteriophage followed method 9224E, “Detection of Coliphages, Single Agar Method” from Standard Methods for the Examination of Water and Wastewater (American Public Health Association) (28), with the following modifications: agar concentration of 1%, 2,3,5-triphenyl tetrazolium chloride added to the tryptone yeast glucose agar (TYGA), RNase control was not added since the phage were predetermined. Survival curves were plotted to determine the UV sensitivity of the phage.
